# Statistics of Breathing—a New Cure for Toothache

**Published:** 1890-01

**Authors:** 


### Statistics of Breathing.

In each respiration an adult inhales one pint of air.

A man respires sixteen to twenty times a minute, or twenty thousand times a day; a child, twenty five to thirty five times a minute.

While standing, the adult respiration is twenty-two; while lying, thirteen.

The superficial surface of the lungs, *i. e.*, of their alveolar space, is two hundred square yards.

The amount of air inspired in twenty-four hours is ten thousand litres (about ten thousand quarts).

The amount of oxygen absorbed in twenty-four hours is five hundred litres (744 grammes); and the amount of carbonic acid gas expired in the same time, four hundred litres (911.5 grammes).

Two-thirds of the oxygen absorbed in twenty-four hours is absorbed during the night hours from 6 P. M. to 6 A. M.

Three-fifths of the total carbonic acid is thrown off in the day time.

The pulmonary surface gives off one hundred and fifty grammes of water daily in the state of vapor.

An adult must have at least three hundred and sixty litres of air an hour.

The heart sends through the lungs eight hundred litres of blood hourly, and twenty thousand litres, or five thousand gallons, daily. The duration of inspiration is five-twelfths, of expiration seven-twelfths, of the whole respiratory act; but during sleep inspiration occupies ten-twelfths of the respiratory period.—*Annals of Hygiene.*

A New Cure for Toothache.—Some one has discovered that a weak galvanic current, which will sometimes cure a toothache, may be generated by placing a silver coin on one side of the gum and a piece of zinc on the other. Rinsing the mouth with acidulated water is said to increase the effect.—*Weekly Med. Review.*

